# Transgenic minipig model of Huntington's disease exhibiting gradually progressing neurodegeneration

**DOI:** 10.1242/dmm.041319

**Published:** 2019-12-12

**Authors:** Taras Ardan, Monika Baxa, Božena Levinská, Miroslava Sedláčková, The Duong Nguyen, Jiří Klíma, Štefan Juhás, Jana Juhásová, Petra Šmatlíková, Petra Vochozková, Jan Motlík, Zdenka Ellederová

**Affiliations:** 1 Laboratory of Cell Regeneration and Plasticity, Institute of Animal Physiology and Genetics, Czech Academy of Science, 27721 Libechov, Czech Republic; 2 Department of Histology and Embryology, Masaryk University in Brno, Faculty of Medicine, 62500 Brno, Czech Republic

**Keywords:** Large animal model, TgHD, Brain, Huntingtin, Neuropathology

## Abstract

Recently developed therapeutic approaches for the treatment of Huntington's disease (HD) require preclinical testing in large animal models. The minipig is a suitable experimental animal because of its large gyrencephalic brain, body weight of 70-100 kg, long lifespan, and anatomical, physiological and metabolic resemblance to humans. The Libechov transgenic minipig model for HD (TgHD) has proven useful for proof of concept of developing new therapies. However, to evaluate the efficacy of different therapies on disease progression, a broader phenotypic characterization of the TgHD minipig is needed. In this study, we analyzed the brain tissues of TgHD minipigs at the age of 48 and 60-70 months, and compared them to wild-type animals. We were able to demonstrate not only an accumulation of different forms of mutant huntingtin (mHTT) in TgHD brain, but also pathological changes associated with cellular damage caused by mHTT. At 48 months, we detected pathological changes that included the demyelination of brain white matter, loss of function of striatal neurons in the putamen and activation of microglia. At 60-70 months, we found a clear marker of neurodegeneration: significant cell loss detected in the caudate nucleus, putamen and cortex. This was accompanied by clusters of structures accumulating in the neurites of some neurons, a sign of their degeneration that is also seen in Alzheimer's disease, and a significant activation of astrocytes. In summary, our data demonstrate age-dependent neuropathology with later onset of neurodegeneration in TgHD minipigs.

## Abstract (precis)

**Summary:** Longitudinal phenotyping of the minipig model for Huntington's disease demonstrates a slow and age-dependent neurodegeneration.

## INTRODUCTION

Huntington's disease (HD) is an inherited progressive neurodegenerative disease without a current effective treatment. It is caused by CAG triplet expansion in exon 1 of the huntingtin gene (*HTT*), which gives rise to mutant huntingtin protein (mHTT). HD patients suffer from involuntary chorea-like movements, poor balance, cognitive dysfunction, emotional disturbances and weight loss. HD manifests typically between 30 and 50 years of age (correlating with CAG repeat size and genetic and environmental modifiers) [Genetic Modifiers of Huntington's Disease (GeM-HD) Consortium et al., 2015; Gusella et al., 2014].

Even though HD is a monogenic disease, the pathogenesis is rather complicated due to the important role of huntingtin protein (HTT) in diverse cellular processes, including transcription, RNA splicing, endocytosis, trafficking, anti-apoptotic processes and cellular homeostasis (Harjes and Wanker, 2003). It is believed that misfolded mHTT undergoes disease-specific enhanced proteolysis leading to mHTT fragmentation (Mende-Mueller et al., 2001). Soluble mHTT monomers, N-terminal fragments and mHTT oligomers, so-called mHTT intermediates of the aggregation pathway, were described as a trigger of cellular dysfunction in the affected tissues (Hoffner et al., 2007; Lajoie and Snapp, 2010).

The most affected organ in HD is the brain; especially vulnerable are the medium-sized spiny neurons in the striatum and the pyramidal cells in the cortex (Zuccato et al., 2010). In addition to the atrophy of medium spiny neurons, white matter atrophy, myelin breakdown and microglia activation are connected to HD (Bartzokis et al., 2007; Paulsen, 2010). Even though the brain pathology appears before the clinical onset of the disease, widespread neuronal loss occurs at a later stage of HD (Rosas et al., 2008).

The primary goal of HD research is to develop disease-modifying treatment that will prevent or postpone the onset and slow the progression of clinical symptoms in HD patients. Unfortunately, several promising therapies with powerful results in HD mouse models failed to be efficient in humans, such as the mitochondrial coenzyme Q10 (coQ10) (Huntington Study Group, 2001; McGarry et al., 2017) and creatine (Hersch et al., 2017). The rodent's small brain size, differences in neuroanatomy relative to humans and short lifespan limit their application for detailed modelling of the pathogenic features of human neurodegenerative diseases. Therefore, large animal models are desired especially for safety, tolerability and efficacy tests of potential therapeutics, and longitudinal studies of HD. To this end, several large animal models have been generated, such as non-human primates, sheep and pigs (Baxa et al., 2013; Jacobsen et al., 2010; Menalled et al., 2009; Uchida et al., 2001; Yan et al., 2018; Yang et al., 2010). The advantages of pigs, especially minipigs, compared with the other models are the relatively large gyrencephalic brain with similar neuroanatomy to humans, a white:grey matter ratio (60:40) comparable to that of humans, adult body weight of 70-100 kg, longer lifespan of 12-15 years, relatively low cost, and fewer ethical problems (Vodička et al., 2005). Moreover, minipigs are easily maintained in controlled conditions and their litter size is usually six to eight piglets, thus providing good experimental groups with similar genetic background.

The transgenic HD minipig (TgHD) model was generated in Libechov, Czech Republic by the use of a lentiviral vector expressing the N-terminal part of the human mHTT (N548-124CAG/CAA) under the control of human *HTT* promoter injected into one-cell embryos (Baxa et al., 2013). Only one copy of the construct was incorporated into the minipig genome on chromosome 1 (1q24-q25), not interrupting any coding sequence (Macakova et al., 2016). Pigs from subsequent generations express human mHTT in all tissues, with the highest levels being detected in the brain and testes (Macakova et al., 2016; Vidinská et al., 2018). Previously, sperm and testicular degeneration, impairments of mitochondrial metabolism and glycolysis, a reduction of DARPP32 (dopamine-regulated neuronal phosphoprotein) and the presence of other markers of neurological phenotype progression were demonstrated (Askeland et al., 2018; Krizova et al., 2017; Macakova et al., 2016; Vidinská et al., 2018).

The TgHD minipig model was proven to be useful in preclinical testing of human HTT-lowering gene therapy, showing widespread vector distribution and considerable HTT lowering (Evers et al., 2018). Several injected TgHD animals and age-matched TgHD non-injected controls from the following longitudinal study are still alive and are being monitored. Therefore, a detailed characterization of the TgHD minipig's phenotype is required to detect the therapeutic effect of HTT lowering as well as of other therapeutic interventions.

Here, we aimed to further characterize the neuropathological phenotype as the TgHD experimental animals age. We examined the brain tissue in terms of ultrastructure, and biochemical and histochemical manifestation of important markers of neurodegeneration at 48 months (4 years) and 60-70 months (5-5.8 years).

## RESULTS

### Genotype- and gender-specific weight loss in TgHD minipigs

**Fig. 1.**
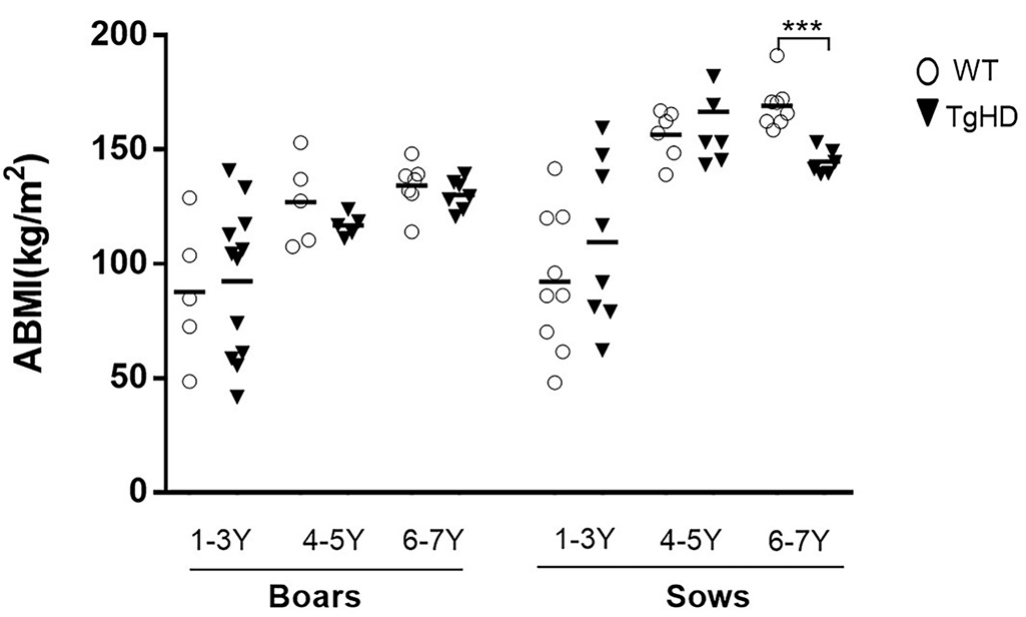
**The animal body mass index (ABMI) measurement of TgHD and WT minipigs of F1 and F2 generations at different ages.** A graph shows ABMIs for sows and boars within three age groups: 1- to 3-year-old (1-3 Y) boars (TgHD *N*=12, WT *N*=5) and sows (TgHD *N*=8, WT *N*=9), 4-5 Y boars (TgHD *N*=5, WT *N*=5) and sows (TgHD *N*=7, WT *N*=6) and 6-7 Y boars (TgHD *N*=7, WT *N*=7) and sows (TgHD *N*=6, WT *N*=8). Student's t-test with Mann–Whitney test. ***P<0.001.

Previously, we investigated the motor and cognitive performance of 48-month-old minipigs and detected a general tendency for reduced performance in all tests with a significant decline in the ability to perform the tunnel test in the TgHD minipigs (Askeland et al., 2018). Because motor and cognitive phenotype is connected with weight loss, we also measured the animal body mass index (ABMI), a weight correlated by height and length of the animal. Animals at the age of 1, 2, 3, 4, 5, 6 and 7 years were measured. In order to have enough animals in each group to perform statistical analysis, we pooled ages 1-3.9, 4-5.9 and 6-7.9 years (Fig. 1A). The ABMI values of boars increase up to the age of 4 years. From the age of 4 years, the ABMI of boars remains on the same level. The ABMI of both wild-type (WT) and TgHD sows increases up to the age of 4 years. From the age of 5 years, the ABMI of TgHD sows decreases, while the change in AMBI of WT sows is minimal. While just a slight non-significant decrease was revealed in the ABMI of TgHD compared to WT boars at 6-7 years, a significant decrease was measured in 6- to 7-year-old TgHD sows (6 years: *P*=0.0286; 7 years: *P*=0.0357; 6-7 years: *P*=0.0002) in comparison to the WT controls.

### mHTT intermediates of the aggregation pathway accumulate in an age- and brain-region-specific manner in TgHD minipigs

**Fig. 2.**
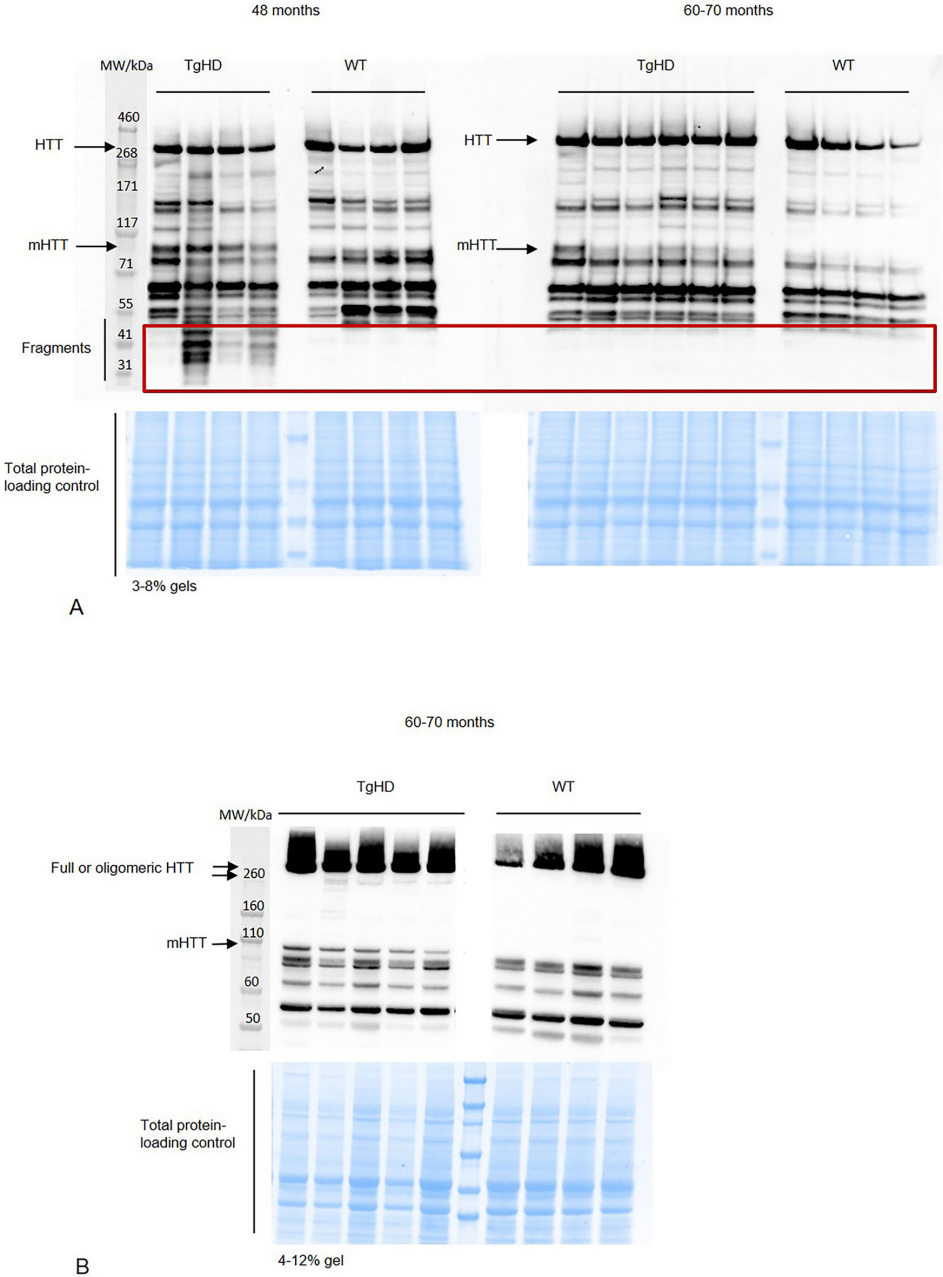
**Western blot analysis of HTT forms.** (A) Detection of fragmented HTT in putamen from 48- and 60- to 70-month-old minipigs using 3-8% gels and EPR-5526 anti-HTT antibody. (B) Detection of oligomeric forms of HTT in putamen from 60- to 70-month-old minipigs using 4-12% gels and EPR-5526 anti-HTT antibody. Representative blots from different TgHD and WT animals are shown.

We suppose that the changes between WT and TgHD brain tissue are caused by the expression of mHTT. The expression of the N-terminal part of human mHTT (∼110 kDa) in the TgHD minipigs and its absence in WT minipigs was confirmed at all ages (from 1 to 4 years) and in different generations by us previously (Askeland et al., 2018; Baxa et al., 2013; Vidinská et al., 2018). Here, we evaluated the expression of mHTT (∼110 kDa), endogenous HTT (∼350 kDa) and its forms by western blot using an HTT-specific antibody in the brain of 48-month-old and 60- to 70-month-old minipigs. We detected expression of mHTT, and its several smaller fragments, mainly in 48-month-old TgHD putamen samples (Fig. 2A). Using a different percentage gel (4-12%) we also detected smears with two bands at the high molecular weight in 60- to 70-month-old TgHD putamen samples, presumably showing oligomeric structures (Fig. 2B). Based on this and our previous results, we conclude that the forms of HTT change during ageing.

For the identification and localization of HTT and possible inclusions/aggregates by immunohistochemistry, the following commercially available primary antibodies were used: BML-PW0595, EPR5526 and MW8. The majority of HTT expression was localized in the spiny neurons of the striatum and in the cortical pyramidal neurons. MW8 antibody was used to reveal potential aggregates. Even when using this antibody, we detected a few structures with different diameters in all TgHD basal ganglia that were comparable to aggregates observed in HD human brain. Very similar aggregate formations were also observed in WT basal ganglia. Therefore, we were not able to draw a definitive conclusion from these results. Since a recent manuscript (Jansen et al., 2017) shows that the percentage of neurons having aggregates in post-mortem HD patient brain samples does not exceed 0.3%, it is possible that the aggregates in TgHD brain were under the detection limit.

### Age- and genotype-specific shift of characteristic markers of neurodegeneration (cellular damage)

In order to recognize specific markers of cell damage, we stained brain coronal sections of 48- and 60- to 70-month-old minipigs with anti-Iba1 (ionized calcium-binding adapter molecule 1), anti-GFAP (glial fibrillary acidic protein) and anti-DARPP32 antibodies.

**Fig. 3.**
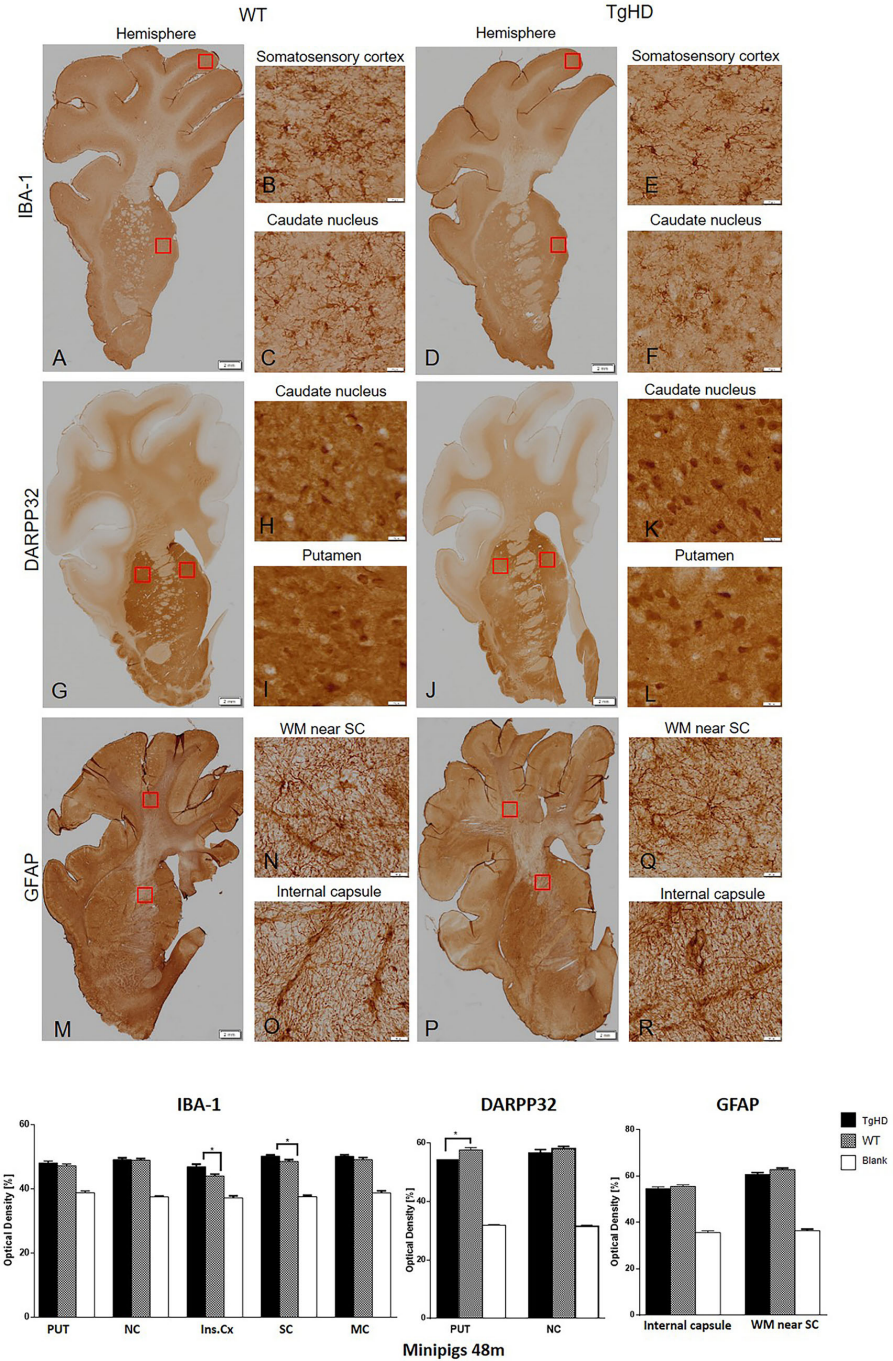
**Immunohistochemical investigation of expression of IBA-1, DARPP32 and GFAP in the brain sections of 48-month-old animals.** IBA-1 (A-F); DARPP32 (G-L); GFAP (M-R). The graph below shows that image analysis of the immunohistochemical staining demonstrated significantly increased IBA-1 expression in the insular and somatosensory cortex, and significantly decreased DARPP32 expression in putamen of TgHD animals. **P<0.05; PUT, putamen; NC, caudate nucleus; Ins.Cx, insular cortex; SC, somatosensory cortex; MC, motor cortex; WM, white matter; Blank, staining without primary antibody. Scale bars: hemispheres, 2 mm; enlargements of brain structures, 50 µm.

At 48 months, levels of ionized calcium-binding adapter molecule 1 (Iba1), a specific marker of microglia and their activation state, were evaluated. The semi-quantitative image analysis of Iba1 immunostaining showed higher, statistically significant expression only in the insular (*P*=0.0117) and somatosensory (*P*=0.0414) cortex of 48-month-old TgHD minipigs compared to WT (Fig. 3). Activated astrocytes and their proliferation activity were determined by GFAP staining. GFAP expression is required for normal function of fibrous astrocytes (Liedtke et al., 1996). Of note, the majority of protoplastic astrocytes do not express enough GFAP to stain positive with routine immunohistochemical (IHC) methods (Chen and Swanson, 2003; Walz, 2000), and consequently most astrocytes in grey matter are GFAP-negative with routine staining. This corresponds to our finding in which astrocytes were clearly stained in the white matter, whereas the grey matter structures were less intensively labelled. The image analysis of GFAP staining demonstrated no significant changes between WT and TgHD minipigs in the 48-month-old brain substructures of interest (Fig. 3). Last, we examined the expression of DARPP32 in minipig striatum and cortex. DARPP32 is the selective marker of striatal medium spiny neurons and a potent inhibitor of protein phosphatase 1, which plays an important role in dopaminergic and glutamatergic signalling. Neurons in the striatum exhibited very strong DARPP32 staining, whereas neurons located in the cortex had a weaker signal. The results of image analysis of DARPP32 labelling showed a reduced level of expression in the striatum with a significant relevance in putamen (*P*<0.05) of TgHD compared to WT animals (Fig. 3). Since DARPP32 is a selective marker of striatal medium spiny neurons, our finding suggests the loss of function of these neurons with consequences on dopaminergic signalling in the striatum of TgHD minipig brain.

**Fig. 4.**
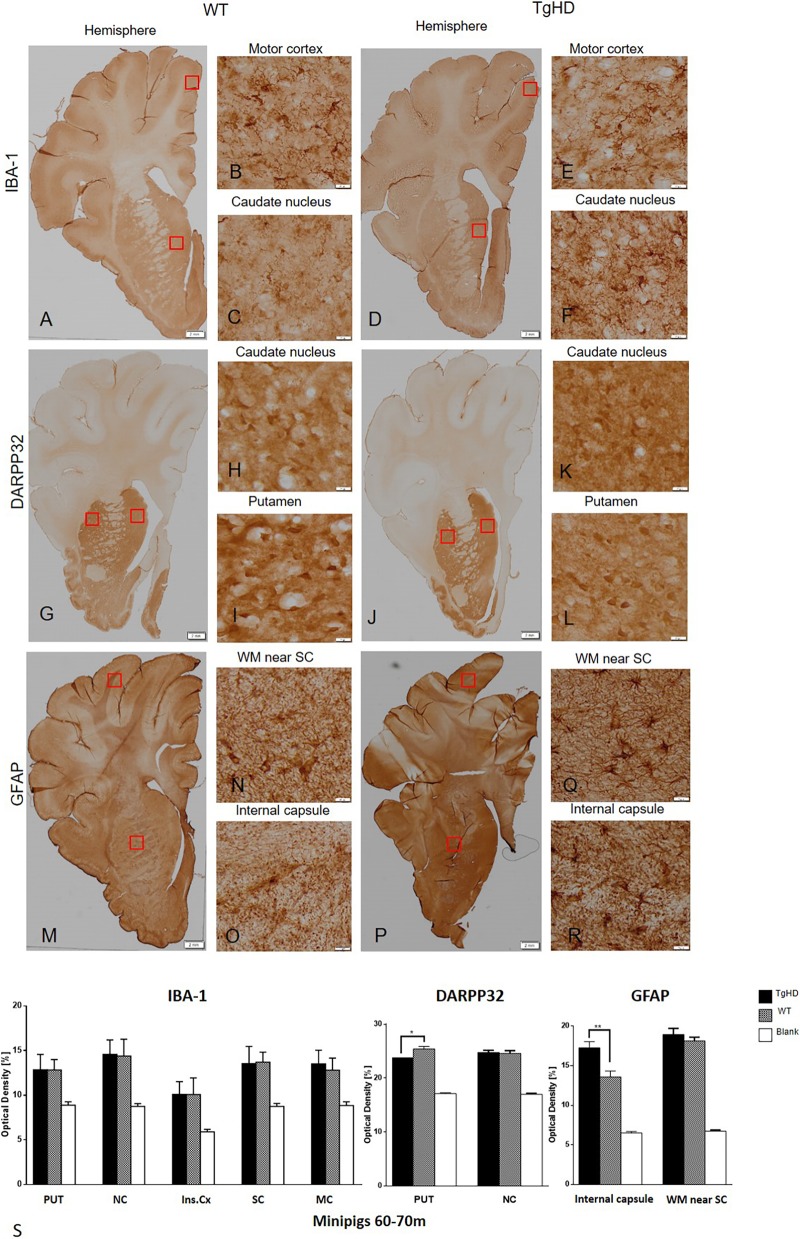
**Immunohistochemical investigation of expressions of IBA-1, DARPP32 and GFAP in the brain sections of 60- to 70-month-old animals.** IBA-1 (A-F); DARPP32 (G-L); GFAP (M-R). (S) Image analysis of the immunohistochemical staining demonstrated significantly increased GFAP expression in the internal capsule and significantly decreased DARPP32 expression in putamen of TgHD animals. *P<0.05; **P≤0.01; PUT, putamen; NC, caudate nucleus; Ins.Cx, insular cortex; SC, somatosensory cortex; MC, motor cortex; WM, white matter; Blank, staining witout primary antibody. Scale bars: hemispheres, 2 mm; enlargements of brain structures, 50 µm.

At 60-70 months, staining of Iba1 in IHC brain sections indicated only a slightly increased expression in the motor cortex of TgHD minipig brain; however, the image analysis of Iba1 immunostaining did not show any statistically significant differences between WT and TgHD minipigs (Fig. 4). Unlike in 48-month coronal sections, we detected significantly increased expression of the astrocyte marker GFAP in the internal capsule (*P*<0.01) and also increased (non-significantly) expression in the somatosensory cortex in TgHD 60- to 70-month-old minipigs compared to WT (Fig. 4). The image analysis of DARPP32 labelling consistently showed a significantly reduced level of its expression in the putamen (*P*=0.02) of TgHD compared to WT, similar to those from 48-month-old animals (Fig. 4).

**Fig. 5.**
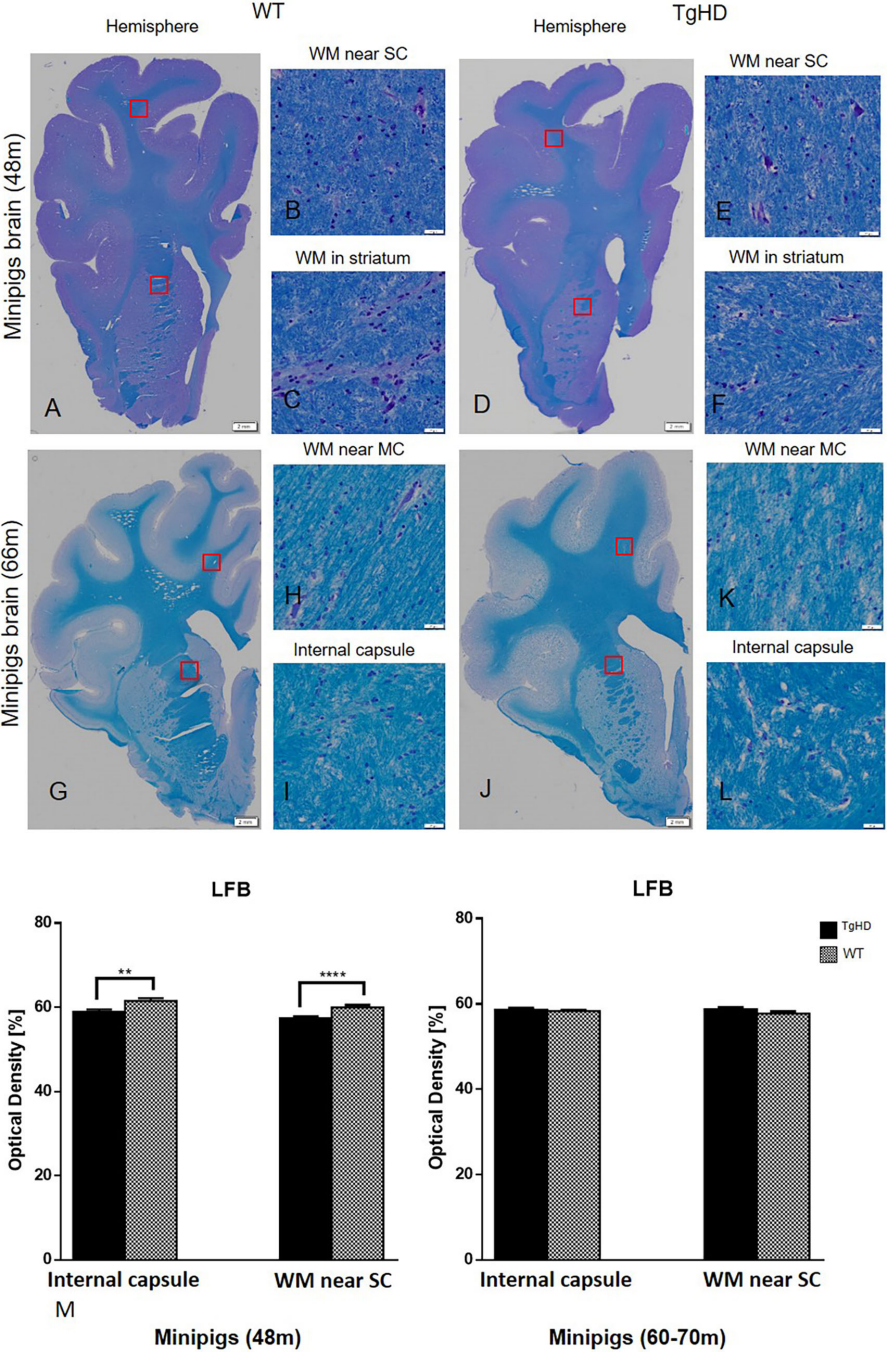
**Histochemical staining of pig brains and quantification of myelinization in white matter.** (A-L) Luxol Fast Blue (LFB) histochemical staining of pig brains. (M) Quantification of myelinization in white matter on minipig coronal brain sections of 48- and 60- to 70-month-old animals. Significantly decreased intensity of myelin staining was detected in the internal capsule (E) and somatosensory cortex (F) of 48-month-old TgHD animals. **P≤0.01; ****P≤0.001. No changes of myelinization were detected in 60- to 70-month-old minipigs (G-M). WM, white matter; SC, somatosensory cortex; MC, motor cortex. Scale bars: hemispheres, 2 mm; enlargements of brain structures, 50 µm.

For the histochemical demonstration of myelin, Luxol Fast Blue staining was employed. Results of this staining showed significantly decreased myelination of nerve fibres in the internal capsule (*P*=0.003) and in the subcortical white matter (*P*<0.0001) of TgHD minipigs in comparison to WT at 48 months (Fig. 5), but no change in older minipigs (60-70 months) compared to WT.

### Altered ultrastructure and cellular loss in the brain of 60- to 70-month-old TgHD minipigs

**Fig. 6.**
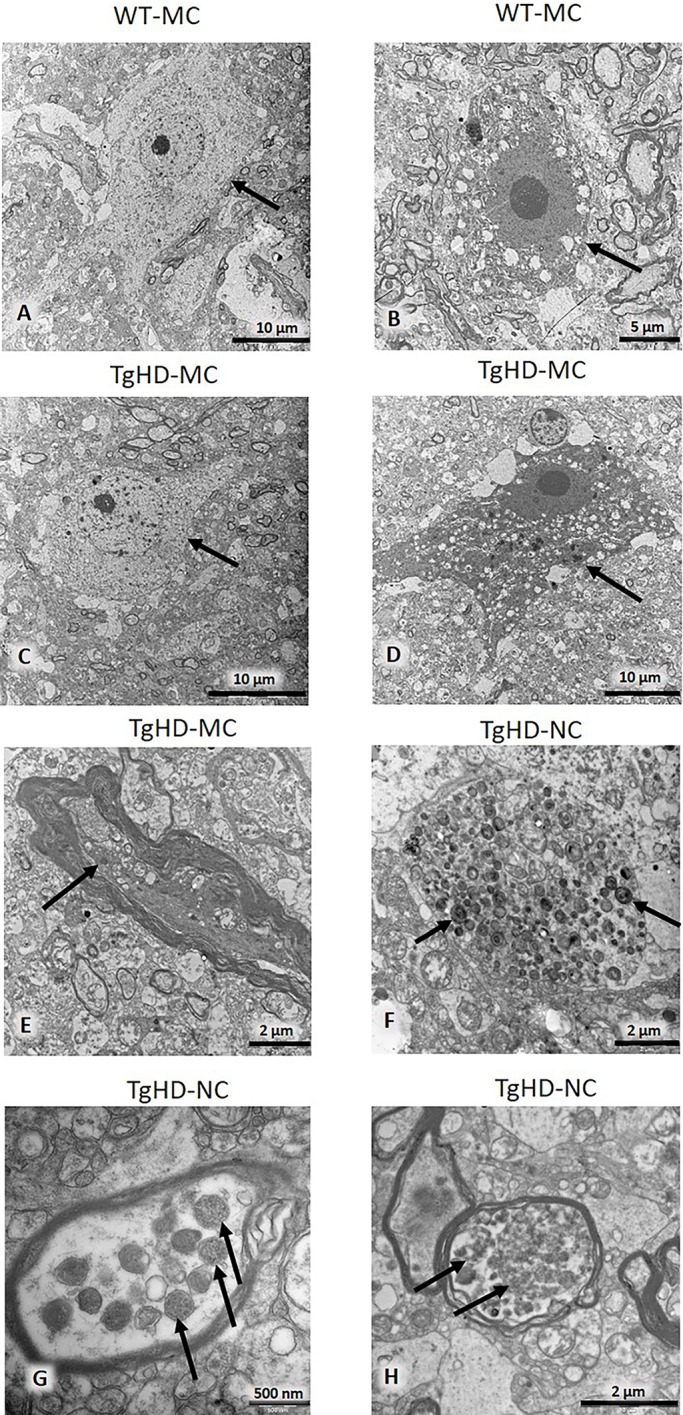
**Electron microscopy of motor cortex and caudate nucleus.** Arrows indicate light (A,C) and dark (B,D) neurons. Dystrophic neurite (E). Accumulation of multilamellar bodies in unmyelinated neuronal process (F). Dense bodies in the myelinated process are probably remnants of degenerated mitochondria (G). Autophagic vacuoles in a myelinated process (H). MC, motor cortex; NC, caudate nucleus.

To visualize the ultrastructure of the striatum and cortex, all 60- to 70-month-old brain sections were processed for transmission electron microscopy (TEM). An initial observation pointing to signs of degeneration was the presence of light and dark neurons, assuming the dark ones to be actually degenerating as previously described in HD mice (Turmaine et al., 2000). However, these neurons were found in TgHD as well as in WT samples, and, referring to the literature, the dark cells were eventually evaluated as artefacts that arose during tissue manipulation and processing (Jortner, 2006). Previously, TEM analysis of HD mice revealed inclusions of mHTT in the nuclei as well as in the cytoplasm of the neurons (dark and light), and in the glia (Davies et al., 1997). But, just as in IHC analysis, we could see a few inclusion-like structures in TgHD as well as in WT samples. There were perhaps a few more inclusions in the TgHD samples of the cortex, which could possibly be interpreted as lipofuscin. We also examined the shape and structure of the nucleus. In TgHD neurons, the folds of the nucleus were seen more often, but sometimes they were seen also in WT. However, clusters of structures accumulating in the neurites of some neurons, which are probably a sign of their degeneration, were detected only in TgHD samples (Fig. 6). These structures are morphologically identical to those detected in Alzheimer's disease (Nixon et al., 2005). Neuronal bodies are not affected, but neurites reveal a mild neurodegeneration of TgHD brain.

**Fig. 7.**
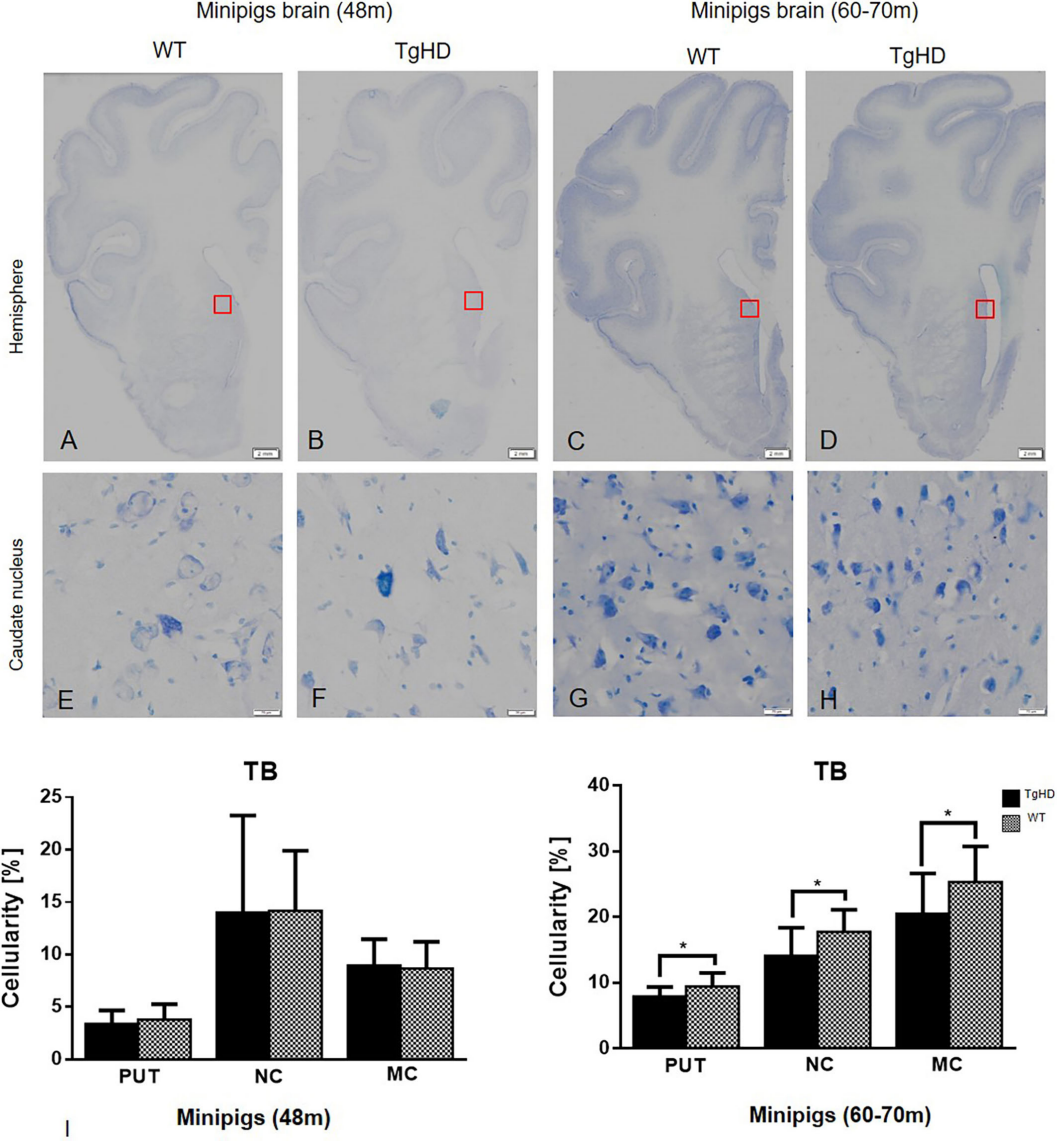
**Toluidine Blue histochemical staining and quantification of cellularity.** Hemispheres (A-D); caudate nucleus (E-H). (I) Quantification of cellularity in striatum and motor cortex of minipig brain sections of both 48- and 60- to 70-month-old animals using image analysis methods. Significantly decreased cellularity was detected in the putamen (PUT), caudate nucleus (NC) and motor cortex (MC) of TgHD 66-month-old animals. *P≤0.05. TB, Toluidine Blue. Scale bars: hemispheres, 2 mm; enlargements of brain structures, 50 µm.

Further, we employed Toluidine Blue staining for the determination of cellularity in WT and TgHD pig basal ganglia and cortex. This method for measuring cellularity was chosen due to the thickness of our cryosections (40 µm) (Gutiérrez et al., 2012). The changes in cellularity were measured on segmented images using an image analysis method, and the cellularity was calculated as the percentage of nuclei staining in the selected region of interest (ROI). Results of statistical analysis, where unpaired Student's *t*-test was applied, showed no significant differences in cellularity between WT and TgHD basal ganglia at 48 months. However, it showed significantly decreased cellularity of TgHD in both striatal areas (caudate nucleus, *P*=0.0198; putamen, *P*=0.0245) and motor cortex (*P*=0.0355) at 60-70 months (Fig. 7). These results indicate genotype- and age-specific loss of cells in TgHD minipig brains.

## DISCUSSION

The TgHD minipig is an important biomedical model primarily designated for testing therapeutic interventions. It can overcome the gap between rodent models and human patients to gain more preliminary knowledge before proceeding with demanding and expensive clinical trials. For this reason, an extensive phenotypical characterization of the TgHD minipig model is highly warranted. The previous characterization showed locomotor functional decline together with genotype-specific effects on mitochondrial DNA (mtDNA) damage, mtDNA copy number and markers of a metabolic alteration that manifest in a progressive neuropathology at 48 months (Askeland et al., 2018). In the present study, we extended our observations and tested older animals for weight loss. Weight loss is a hallmark of HD progression, and the decrease in patients' body mass index (BMI) is associated with functional, motor and cognitive decline (Aziz et al., 2018). Accordingly, we found a significant decrease in the ABMI of 6- to 7-year-old sows and a slight non-significant decrease was revealed in the ABMI of TgHD boars compared to WT boars at the same age (Fig. 1). The reason why we could detect a greater change in ABMI of TgHD versus WT sows compared to boars is due to their body constitution. Sows are generally heavier and tend to have a greater appetite; therefore, a defect in food intake is more easily detected. Lower ABMIs in older TgHD animals is also consistent with our previous data of a perturbed mitochondrial phenotype in TgHD minipig muscle tissue starting at 36 months, prior to the development of mitochondrial ultrastructural changes and locomotor impairment beginning at the age of 48 months (Rodinova et al., 2019).

There is strong evidence that HTT is fragmented in affected individuals (Bates et al., 2015) and the N-terminal mHTT fragments accumulate with disease progression, translocate into the nucleus and cause aberrant protein interaction leading to cellular dysfunction (Benn et al., 2005; Graham et al., 2006; Saudou et al., 1998). mHTT also forms aggregates that were initially described as being the toxic trigger in HD (Davies and Scherzinger, 1997). However, later studies suggest also a protective role of aggregates, as they reduce the level of the toxic soluble protein (Miller et al., 2010; Saudou et al., 1998). Thus, soluble intermediates of the aggregation pathway, oligomers forming from mHTT fragments, are described as the most reactive harmful species (Truant et al., 2008). We previously reported tissue-specific and age-correlated progressive HTT fragmentation in different tissues collected from animals up to 24 months (Vidinská et al., 2018). Here, we show severe mHTT fragmentation at 48 months but less fragmentation occurring at 60-70 months (Fig. 2A). This can be explained by the aggregation process, where fragments at a certain point start to form oligomeric structures (Fig. 2B). This age-dependent process has been previously seen in R6/2 and knock-in HD mice (Sathasivam et al., 2010).

In this study, we also demonstrate the age-related changes in markers of neurodegeneration in TgHD brains at two time points, 48 months and 60-70 months. Reduction of DARPP32, an integrator of neurotransmission, has been described in different HD models well before the onset of the behavioural phenotype (Heng et al., 2007; Woodman et al., 2007). Also in our TgHD minipig model, we previously reported downregulation of DARPP32 at 16 and 24 months (Baxa et al., 2013; Vidinská et al., 2018). Consistently, here we report the downregulation of DARPP32 at 48 months as well as at 60-70 months.

We also show microglial activation at 48 months. This result is in line with microglial activation in brain sections of 24-month-old TgHD minipigs (Vidinská et al., 2018), together with decreased levels of IFNα and IL-10 and increased levels of IL-8 and IL-1β in the microglial secretome in TgHD compared to WT controls (Valekova et al., 2016). The increased levels of IL-8 and IL-1β were also found in plasma of pre-manifest HD patients and were linked to increased central microglial activation (Politis et al., 2015). It was recently revealed that the activated microglia induce the production of A1 astrocytes (Liddelow et al., 2017). In the present study, we used GFAP as a marker of astrocyte activation, and we did not detect activation of astrocytes at 48 months but observed a significant increase of activation at 60-70 months, which could be an effect of high microglial activation at 48 months. It was shown that A1 astrocytes fail to support neuronal survival; in contrast, they can trigger neuronal degeneration (Liddelow et al., 2017). Their increased number was demonstrated in HD as well as in other neurodegenerative diseases (Hinkle et al., 2019). However, the higher presence of A1 astrocytes specifically was not measured in TgHD minipigs. Therefore, it is just an assumption that our detection of activated astrocytes reflects a higher production of harmful A1 astrocytes, and it needs to be further validated.

Additionally, we detected demyelination at 48 months similarly to in our previous study, where we examined brain sections from 24-month-old TgHD animals compared to WT (Vidinská et al., 2018). Also, in different mouse models, the demyelination occurs before neurodegeneration (Teo et al., 2016). Activated microglia expressing proinflammatory mediators damage oligodendrocytes and consequently cause demyelination of white matter (Peferoen et al., 2014). It is interesting that both microglia activation and demyelination were significant at 48 months but not at 60-70 months, at which point astrocyte activation takes place.

As previously discussed, no genotype-specific aggregates were found in the brains of TgHD minipigs by IHC. However, the TEM analysis revealed TgHD-specific inclusions in the axons of some neurons (Fig. 6). Inclusions in axons were also detected in HD mice and associated with axonal degeneration (Li et al., 2001). Inclusions can block axonal transport and thus contribute to the degeneration of mitochondria and other organelles, and ultimately lead to neuronal degeneration. However, it is also possible that mHTT directly binds to synaptic vesicles and affects synaptic transmission before forming large aggregates (Usdin et al., 1999). We also found age- and genotype-related cellular loss in basal ganglia and cortex (Fig. 7). Cellular degeneration particularly in basal ganglia and cortex is the hallmark of HD progression (Zuccato et al., 2010). Our finding of axonal inclusions together with the age-dependent cellular degeneration is one of the main findings of this study and shows slow but progressive neurodegeneration in the TgHD minipig model with the N-terminal part of human mHTT. The slow progression observed in this model is surprising since the triplet repeat length is 124, thus modelling a juvenile form of the disease. It is possible that the slow progression is due to the CAG/CAA mix of the repeat region of mHTT. This design aimed for better stability of the construct when generating this TgHD minipig model in 2008 (Baxa et al., 2013). Nevertheless, later on it was revealed that there is a dramatic striatal-specific somatic repeat expansion in HD patients, causing the striatal cells to be more vulnerable to the effect of mHTT (Swami et al., 2009). Also, the new knock-in HD-150Q porcine model containing only CAG repeats revealed somatic as well as germline CAG instability together with a robust phenotype (Yan et al., 2018). Importantly, the slow progression of the TgHD minipig model and the availability of disease biomarkers can be instrumental in the evaluation of HD treatment efficacy. For example, it could help to assess the treatment efficacy in the ongoing (application in July 2017) AAV5-miHTT longitudinal HD preclinical study.

## MATERIALS AND METHODS

### Minipig material and sample collection

Transgenic minipigs expressing the N-terminal part of human mHTT were studied. The genotype was determined by PCR according to Baxa et al. (2013) from DNA isolated from minipig skin biopsies after weaning. TgHD minipigs at 48 months old (*n*=6) and their WT controls (*n*=6), and 60- to 70-month-old TgHD minipigs (*n*=6) and their WT controls (*n*=4), from F3 generations were perfused under deep anaesthesia with ice-cold PBS. Various tissues were isolated and stored after snap freezing in liquid nitrogen. The right hemisphere of each perfused brain was directly fixed for immunohistochemistry. The entire study was carried out in agreement with the Animal Care and Use Committee of the Institute of Animal Physiology and Genetics, under the Czech regulations and guidelines for animal welfare and with the approval of Czech Academy of Sciences, protocol number: 53/2015.

### The body mass index calculation

Animals were weighed regularly at the same hour of the day. Only animals from F1 and F2 generations were used. Their body mass indexes (ABMIs) were calculated as follows: ABMI=*m*/*h*×*l*, where *m*=weight of animal, *h*=height of animal at the withers, *l*=length of animal from withers to tailbone. The results were evaluated using GraphPad Prism 6 by *t*-test/Mann–Whitney test.

### SDS-PAGE and western blot

Tissue samples were homogenized in liquid nitrogen using a mortar and lyzed in RIPA buffer (150 mM NaCl, 5 mM EDTA pH 8, 0.05% NP-40, 1% sodium deoxycholate, 0.1% SDS, 1% Triton X-100, 50 mM Tris-HCl pH 7.4, inhibitors of phosphatases and proteases), sonicated for 15 min, and centrifuged at 20,000 ***g***, for 15 min, at 4°C. Samples (10 µg of total protein) were loaded onto 3-8% or 4-12% Tris-acetate gel (#EA03758, LifeTech) and run at 150 V. Gel was transferred onto nitrocellulose membrane, blocked in 5% skimmed milk and probed overnight with anti-HTT antibody diluted in 5% milk (EPR5526, Abcam, 1:3000) at 4°C. Memcode protein staining (LifeTech) was used for normalization of loading. Secondary antibody conjugated with HRP (anti-mouse, #711-035-152, Jackson ImmunoResearch, 1:10,000 or anti-rabbit, #711-035-152, Jackson ImmunoResearch, 1:10,000) was used. The signal was revealed by chemiluminiscence (ECL, #28980926, APCzech) and detected by The ChemiDoc XRS+system (Bio-Rad).

### Immunohistochemistry

**Fig. 8.**
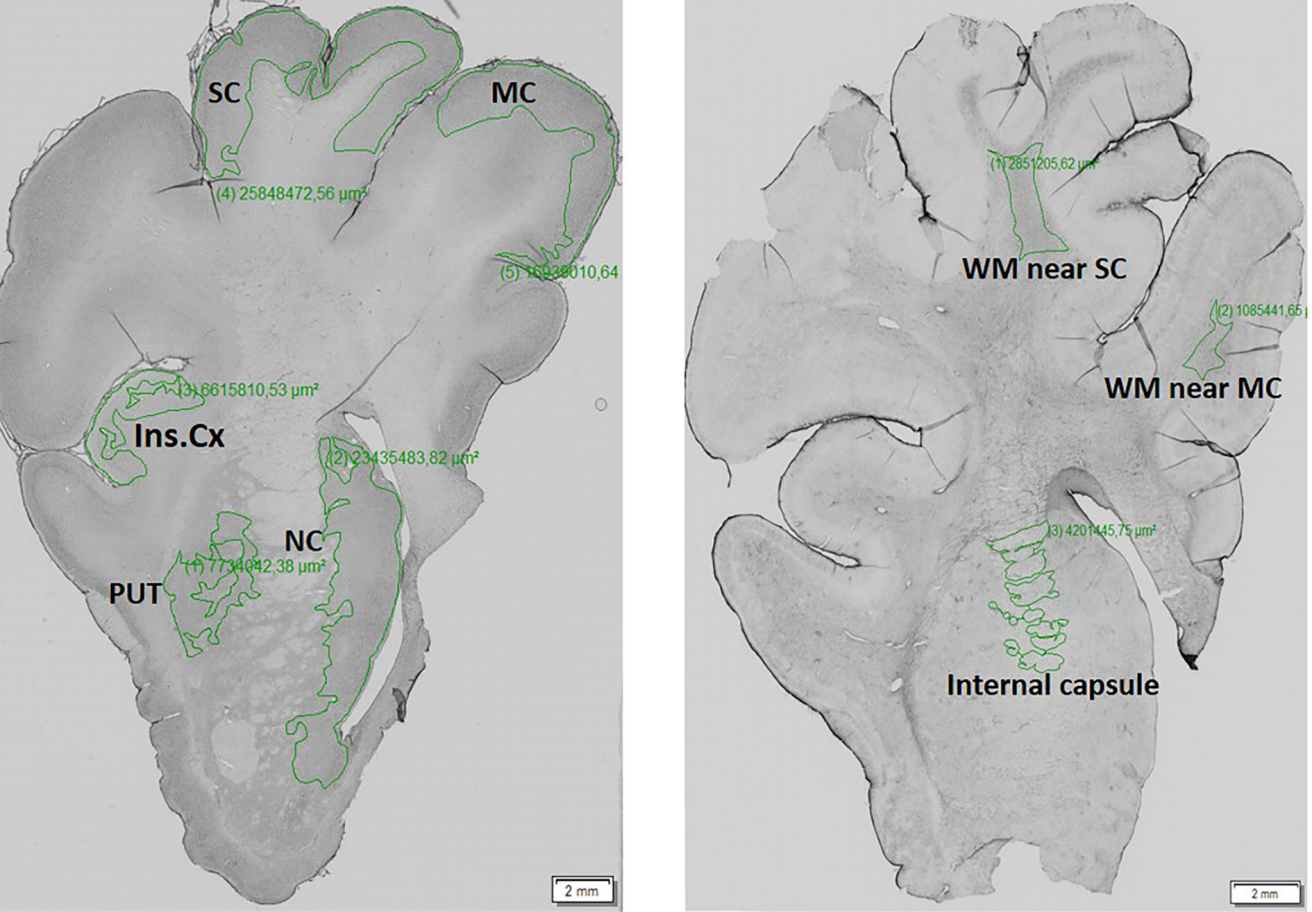
**Brain regions that underwent densitometric measurement of the intensity staining**. The manually selected areas of the porcine brain hemisphere (left) such as motor cortex (MC), somatosensory cortex (SC), insular cortex (Ins.Cx), putamen (PUT) and nucleus caudate (NC), and white matter (WM; right) internal capsule, and WM near the SC and MC, which underwent a densitometric measurement of the intensity staining. The evaluated regions of interest of these brain areas were marked out by a green line.

The right hemisphere from each animal was fixed in 4% paraformaldehyde for 24 h and then cryoprotected with 30% sucrose containing 0.01% sodium azide. Frozen coronal sections were prepared using tissue-freezing medium (Leica, 14020108926). The free-floating sections (three per animal) of a thickness of 40 µm were sequentially treated with formic acid, 0.3% hydrogen peroxide in MetOH, and blocking serum to unmask antigens and reduce endogenous peroxidases and unspecific binding of antibodies. The sections were incubated with the following commercially available primary antibodies diluted in 5% milk (all 1:250) at 4°C: anti-Iba1 (AIF1, Synaptic System), anti-GFAP (G3893, Sigma-Aldrich), anti-DARPP32 (ab40801, Abcam), anti-HTT [BML-PW0595, Enzo Life Science; EPR5526, Abcam; and MW8, AB528297, Developmental Studies Hybridoma Bank, (Iowa City, IA, USA)]. The specificity of primary antibodies was verified by western blot and/or comparative immunohistochemistry of mouse WT and TgHD (R6/2, 12 weeks old) brain sections in the previous study. After washing, sections were incubated with biotinylated donkey anti-rabbit or sheep anti-mouse secondary antibody (both 1:400, Amersham, Buckinghamshire, UK) followed by incubation with avidin-peroxidase complex (1:400, Sigma-Aldrich). The labelled sections by peroxidase were developed with DAB tablets (#4170, Kementec Diagnostics). The specificity of secondary antibodies was confirmed by using negative controls. The evaluation and quantification of immunoreactivity was performed using a densitometry measurement of staining by image analysis software VS-Desktop (Olympus, Tokyo, Japan) and ImageJ (Rasband, W.S., US National Institutes of Health, Bethesda, MD, USA). According to the 3D-view model of pig brain (from programme 3D Slicer; slicer.org) optical sections were divided into substructures: basal ganglia (caudate nucleus, putamen) and cortex (motor and somatosensory and insular), in which the mean intensity was measured (Fig. 8). For statistical analysis a one-way ANOVA test with Tukey's multiple-comparison post-test was employed using GraphPad PRISM software (GraphPad Software, San Diego, CA, USA).

### Histochemical examination of brain tissue

For histochemical demonstration of myelin, Luxol Fast Blue staining was employed. Toluidine Blue staining was used for the determination of cellularity in WT and TgHD pig caudate nucleus. The changes in cellularity were measured on segmented images using an image analysis method and the cellularity was calculated as percentage of nuclei staining in the selected ROI. This densitometry method for cellularity measurement was adopted by Gutiérrez et al. (2012). Unpaired Student's *t*-test was applied for statistical evaluation.

### Electron microscopy (EM)

Small blocks of motor cortex and striatum were fixed in 300 mM glutaraldehyde (Sigma-Aldrich) in 100 mM cacodylate buffer for 2 h at room temperature (RT), washed in the same buffer and post-fixed in 40 mM osmium tetroxide (Polysciences) in 100 mM cacodylate buffer for 1 h at RT. After rinsing in cacodylate buffer and dehydration in ethanol, the samples were embedded in araldite resin (Durcupan ACM, Sigma-Aldrich). Sections (60 nm thick) were cut using a Leica EM UC6 ultramicrotome, and were stained with uranyl acetate and lead citrate. Sections were examined under an FEI Morgagni 268D electron microscope (FEI Company, The Netherlands) at 70 kV.

This article is part of a special collection ‘A Guide to Using Neuromuscular Disease Models for Basic and Preclinical Studies’, which was launched in a dedicated issue guest edited by Annemieke Aartsma-Rus, Maaike van Putten and James Dowling. See related articles in this collection at http://dmm.biologists.org/collection/neuromuscular.
